# M-CSF increases proliferation and phagocytosis while modulating receptor and transcription factor expression in adult human microglia

**DOI:** 10.1186/1742-2094-10-85

**Published:** 2013-07-17

**Authors:** Amy M Smith, Hannah M Gibbons, Robyn L Oldfield, Peter M Bergin, Edward W Mee, Maurice A Curtis, Richard L M Faull, Mike Dragunow

**Affiliations:** 1 Department of Pharmacology and Clinical Pharmacology, The University of Auckland, Private Bag 92019, Auckland 1142, New Zealand; 2 National Research Center for Growth and Development, The University of Auckland, Auckland, New Zealand; 3 Department of Anatomy, The University of Auckland, Auckland, New Zealand; 4 Center for Brain Research, The University of Auckland, Auckland, New Zealand; 5 Lab Plus, Auckland 1023, New Zealand; 6 Auckland City Hospital, Auckland 1023, New Zealand

**Keywords:** Phagocytosis, Human glial culture, Microglial activation, Morphology, PU.1

## Abstract

**Background:**

Microglia are the primary immune cells of the brain whose phenotype largely depends on their surrounding micro-environment. Microglia respond to a multitude of soluble molecules produced by a variety of brain cells. Macrophage colony-stimulating factor (M-CSF) is a cytokine found in the brain whose receptor is expressed by microglia. Previous studies suggest a critical role for M-CSF in brain development and normal functioning as well as in several disease processes involving neuroinflammation.

**Methods:**

Using biopsy tissue from patients with intractable temporal epilepsy and autopsy tissue, we cultured primary adult human microglia to investigate their response to M-CSF. Mixed glial cultures were treated with 25 ng/ml M-CSF for 96 hours. Proliferation and phagocytosis assays, and high through-put immunocytochemistry, microscopy and image analysis were performed to investigate microglial phenotype and function.

**Results:**

We found that the phenotype of primary adult human microglia was markedly changed following exposure to M-CSF. A greater number of microglia were present in the M-CSF- treated cultures as the percentage of proliferating (BrdU and Ki67-positive) microglia was greatly increased. A number of changes in protein expression occurred following M-CSF treatment, including increased transcription factors PU.1 and C/EBPβ, increased DAP12 adaptor protein, increased M-CSF receptor (CSF-1R) and IGF-1 receptor, and reduced HLA-DP, DQ, DR antigen presentation protein. Furthermore, a distinct morphological change was observed with elongation of microglial processes. These changes in phenotype were accompanied by a functional increase in phagocytosis of Aβ_1-42_ peptide.

**Conclusions:**

We show here that the cytokine M-CSF dramatically influences the phenotype of adult human microglia. These results pave the way for future investigation of M-CSF-related targets for human therapeutic benefit.

## Background

Microglial cells are brain-resident immune cells that have many protective roles but can also contribute to neurological disease processes. The functional phenotype of microglia depends on the cell types and specific activating factors in their surroundings, and for this reason microglia are said to have an adaptive or acquired phenotype, reflecting their response to a collection of external signals [1]. Secreted small molecules, such as cytokines and growth factors, allow microglia to communicate with each other, and with other immune and brain cells which express these receptors [2]. In this way microglia can be recruited to sites of injury, where they can respond by ameliorating the damage [3,4]. However, there have also been numerous reports of detrimental microglial activity in response to injury, and neuroinflammation is thought to play a major role in pathogenesis of many neurological disorders, including epilepsy and Alzheimer’s disease [5-9]. The activation of microglia to a beneficial or detrimental phenotype can be described in terms of inducible protein expression, morphology, and functional outcomes such as cytokine production and phagocytosis [8,10]. Modulating microglial phenotype towards a protective role is a potential strategy for the treatment of many brain disorders and thus the factors influencing microglial activation require further research.

One molecule that can influence microglial phenotype is the cytokine macrophage colony-stimulating factor (M-CSF). M-CSF is found in the brain and its receptor is expressed by microglia [11,12]. M-CSF is produced by a range of cells in the developing and adult brain [12,13]. M-CSF mRNA and protein have been found to be constitutively expressed by human fetal astrocytes and low levels of M-CSF mRNA and protein were detected in unstimulated microglia cultures [14]. Du Yan *et al*. (1997) have also demonstrated M-CSF expression in neurons of the adult human brain [12].

Signaling through the M-CSF receptor (CSF-1R) is required for the development and differentiation of microglia [15] and M-CSF has been shown to increase division of rodent and human fetal microglia [14,16,17]. M-CSF also has the ability to change microglial morphology [18,19] and influence microglial activation [20,21]. M-CSF signaling has been shown to be dependent on the transcription factor PU.1 [22], a vital myeloid transcription factor expressed by microglia in the adult human brain [23]. Celada *et al*. (1996) found that sense PU.1 expression constructs increased M-CSF-dependent proliferation in mouse bone-marrow macrophages, and antisense PU.1 constructs reduced proliferation in response to M-CSF [24]. They also found that sense PU.1 constructs gave rise to increased M-CSF receptor expression, and it has previously been demonstrated that PU.1 binds to the *c-fms* promoter [25].

While M-CSF is important for normal brain development and function [26], several studies have found abnormal levels of M-CSF associated with neurological diseases. M-CSF was demonstrated to be upregulated in brain tumors [27,28] and a correlation was found between levels of M-CSF and HIV-associated cognitive impairment [29]. Furthermore, within three months of HIV therapy, levels of both M-CSF and viral RNA in the CSF were reduced [29]. Despite Boissonneault *et al*. (2009) reporting beneficial effects of M-CSF on cognitive impairment and Aβ_1-42_ deposition in a mouse model of Alzheimer’s disease, it has been suggested that increased M-CSF expression could contribute to Alzheimer’s disease pathogenesis [12,30]. Lue *et al*. (2001) cultured glia from adult human brains and found that M-CSF was elevated in Alzheimer’s disease compared to non-demented microglia [31]. M-CSF receptor expression in microglia in human brain tissue was upregulated in lesions of Alzheimer’s disease and amyotrophic lateral sclerosis [11]. On the contrary, while microglia are found to be associated with multiple sclerosis lesions, the relative number of microglia expressing M-CSF and its receptor have been found to decrease [32]. M-CSF is therefore considered a key factor in regulating microglial inflammatory responses in the damaged brain.

Despite this comprehensive body of literature, there are several caveats which may prohibit the linking of functional studies to observations in human brain tissue. Although most of the research on M-CSF and microglia has been carried out using rodent cells and models, it is becoming increasingly clear that there are important differences between rodent microglia and their human counterpart [33,34]. Age has also been shown to have an impact on immune cell responses and activation [35-37]. Furthermore, the micro-environment of the brain may affect immune responses in ways that are different to those of the periphery [4] and while the majority of research on M-CSF has been carried out on peripheral immune cells, the effects of M-CSF on adult human microglia have not been fully investigated. We have thus assessed the phenotypic profile of adult human microglia after exposure to M-CSF and found that this cytokine dramatically influences their phenotype and activation state.

## Methods

### Tissue

Autopsy human adult brain tissue was obtained from the Neurological Foundation of New Zealand Human Brain Bank under the University of Auckland Human Subjects Ethics Committee. Biopsy adult human brain tissue was obtained from patients undergoing surgery for intractable temporal lobe epilepsy and was approved by the Northern Regional Ethics Committee with informed consent from all tissue donors. The specimens used in this paper were from confirmed temporal lobe epilepsy cases with varying degrees of mild and moderate to marked mesial temporal sclerosis (neuropathological grade 3 to 4). Patients had taken a range of medications alone or in combination, including phenytoin, tegretol, topiramate, lamotrigine, and sodium valproate. There were no obvious associations between the results of any of the reported experiments and medication use or degree of sclerosis.

### Human glial cell isolation and culture

Glial cells were isolated from adult human brain tissue as previously described [38,39]. Approximately 2 g of middle temporal gyrus white and grey matter was washed twice in Hanks balanced salt solution (HBSS; Ca^2+^ and Mg^2+^ free, Gibco BRL, Carlsbad, CA, USA). The meninges and visible blood vessels were removed. The tissue was then diced into small (approximately 1 mm^3^) cubes and placed in 10 ml/g tissue warm enzyme dissociation mix containing 2.5 U/ml papain (Worthington, Lakewood, NJ, USA) and 10 U/ml DNase (Invitrogen, Carlsbad, CA, USA) in HBSS and incubated for 30 minutes at 37°C with agitation. An equal volume of DMEM/F12 supplemented with 10% FBS, 1% penicillin-streptomycin-glutamine (Gibco BRL, Carlsbad, CA, USA) (final concentrations: penicillin (100 U/ml), streptomycin (100 μg/ml) and L-glutamine (0.29 mg/ml)) was added to the tissue which was then gently triturated. The cell suspension was passed through a 100 μm nylon cell strainer (Becton Dickinson, Franklin Lakes, NJ, USA), centrifuged for 10 minutes at 160 g, the pellet resuspended in 20 ml medium and plated into 75 cm^2^ tissue culture flasks (Nunc, Roskilde, Denmark) which were incubated at 37°C in 95% air/5% CO_2_. After 24 hours, the debris and unattached cells were removed, centrifuged for 10 minutes at 160 g and replated onto the adhered cells for a further 24 hours. Finally, the debris was removed and the cells carefully washed with medium. Cells were cultured for one to two weeks (in the same medium as for isolation) prior to plating for experiments at 50,000 cells/ml on 96-well plates.

### M-CSF treatment

Mixed primary human glial cell cultures were given two concentrations of 25 ng/ml M-CSF at 0 and 48 hours. Total time of M-CSF treatment was 96 hours.

### BrdU proliferation assay

Following 72 hours exposure to 25 ng/ml M-CSF, 10 μM BrdU was added to the cells for 24 hours. Cells were washed twice with PBS to remove excess BrdU and fixed with 4% paraformaldehyde (PFA) for 15 minutes at room temperature (RT). For immunocytochemistry, cells were first incubated with 2 M HCl at 37°C for 30 minutes. Cells were then washed twice in 0.1 M borate buffer (pH 8.5) and three times in PBS before applying anti-BrdU antibody.

### Immunocytochemistry

Cells were fixed in 4% PFA for 15 minutes at RT then washed for 10 minutes with phosphate-buffered saline containing 0.2% Triton X-100 (PBS-T). Antibodies were diluted in immuno-buffer (PBS-T containing 1% goat serum and 0.04% merthiolate). Cells were incubated with primary antibody (see Table 1) overnight at 4°C with gentle rocking. Alexa Fluor-conjugated and biotinylated secondary antibodies (Table 1) were applied for three hours at RT with gentle rocking. For colorimetric protein detection, ExtrAvidin-HRP was applied for two hours at RT followed by 3,3′-diaminobenzidine tetrahydrochloride (DAB) reaction. To label all nuclei, cells were stained with Hoechst 33258 (Sigma-Aldrich, St Louis, MO, USA) for 30 minutes at RT protected from light. Cells were washed in TNE buffer (pH 7.4) containing 10 mM Tris, 200 mM NaCl and 1 mM EDTA prior to, and following, incubation in 20 μM Hoechst 33258 diluted in TNE buffer.

**Table 1:** Antibodies used for immunocytochemistry

**Antibody**	**Company**	**Catalogue #**	**Dilution**
Rabbit anti-PU.1	Cell Signaling	2258	1:500
Mouse anti-CD45	Abcam	ab8216	1:500
Rabbit anti-CSF-1R	Santa Cruz	Sc-692	1:50
Mouse anti-HLA-DP, DQ, DR	Dako	M0775	1:500
Mouse anti-GFAP	Sigma-Aldrich	G3893	1:5000
Mouse anti-Prolyl 4-hydroxylase	Dako	M0877	1:1000
Rabbit anti-DAP12	Santa Cruz	Sc-20783	1:500
Mouse anti-C/EBPβ	Santa Cruz	Sc-7962	1:250
Mouse anti-IGF-1R	Millipore	MAB1120	1:50
Mouse anti-BrdU	Roche	11170376001	1:500
Rabbit anti-Ki67	Dako	A0047	1:500
Goat anti-rabbit IgG Alexa Fluor^®^ 594	Invitrogen	A11012	1:500
Goat anti-mouse IgG Alexa Fluor^®^ 488	Invitrogen	A11001	1:500
Goat anti-mouse IgG Alexa Fluor^®^ 594	Invitrogen	A11005	1:500
Goat anti-rabbit IgG Alexa Fluor^®^ 488	Invitrogen	A11008	1:500
Goat anti-rabbit biotinylated	Sigma-Aldrich	B7389	1:500
Goat anti-mouse biotinylated	Sigma-Aldrich	B7264	1:500
ExtrAvidin-HRP	Sigma-Aldrich	E2886	1:500

### Phagocytosis assays

To evaluate the effect of M-CSF on primary adult human microglial phagocytosis of amyloid-β_1–42_ amino acid peptide (Aβ_1-42_; Bachem, Bubendorf, Switzerland), phagocytosis assays were performed as previously described [39,40]. Following 72 hours exposure to 25 ng/ml M-CSF, 5 μM Aβ_1-42_ was added to the cells for 24 hours. Cells were washed twice with PBS to remove excess Aβ_1-42_ and fixed with 4% PFA for 15 minutes at RT. Thioflavin S was used to visualize phagocytosed Aβ_1-42_[40].

### Quantitative image analysis of cell number, microglial morphology and phagocytosis

Immunocytochemical, phagocytic and morphological observations have been quantified using a Discovery-1 automated fluorescence microscope (Molecular Devices, Sunnyvale, CA, USA) and Metamorph (6.2.6 software, Molecular Devices, Sunnyvale, CA, USA) image analysis system as previously described [40,41]. Results were logged automatically to Microsoft Excel spreadsheets.

For quantification of microglial morphology, the Journal ‘Microglial Shape’ was written in Metamorph. The journal automatically thresholded each image to isolate CD45-positive microglia, then applied the Integrated Morphometry Analysis tools Elliptical Form Factor (length/breadth) and Shape Factor (4πA/P^2^, P = cell perimeter, A = cell area) to determine cell shape.

### Statistical Analysis

Representative data are displayed as mean ± standard error of the mean (SEM). Experiments were replicated with cells from at least six different individuals. Number of replicates (n) for each experiment is indicated in figure legends. Statistical analysis was carried out using *t*-tests and *P* values of < 0.05 were considered statistically significant differences.

## Results

### Adult human microglia express the M-CSF receptor *in vitro*

Primary mixed glial cultures containing microglia were prepared from biopsy and autopsy adult human brain tissue as previously described [38,39].

**Figure 1:**
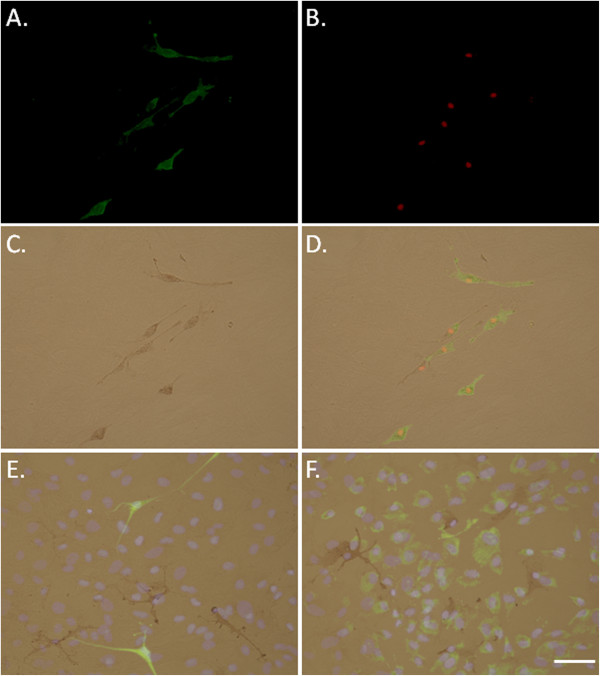
**The macrophage colony-stimulating factor (M-CSF) receptor (CSF-1R) is expressed by adult human microglia *in vitro*. (A)** Adult human microglia in culture labeled for CD45 cell surface protein (green). **(B)** Same field of view showing microglial expression of PU.1 transcription factor (red). **(C)** Brightfield image showing 3,3′-diaminobenzidine tetrahydrochloride (DAB)-labeling of CSF-1R in the same field of view. **(D)** Overlay of images A, B and C demonstrating co-localization of CSF-1R with microglial markers CD45 and PU.1. **(E)** CSF-1R (brown) is not co-expressed by glial fibrillary acid protein (GFAP)-positive astrocytes (green) in mixed glial cultures (all nuclei labeled with Hoechst, blue). **(F)** CSF-1R (brown) is not expressed by prolyl-4-hydroxylase-positive fibroblast-like leptomeningeal cells (green) in mixed glial cultures (all nuclei labeled with Hoechst, blue). Scale bar = 50 μm.

In mixed cultures of adult human glial cells, the M-CSF receptor (CSF-1R) was expressed by microglia. We found that protein expression of CSF-1R coincided with microglial cell surface protein CD45 (leukocyte common antigen) and microglial transcription factor PU.1 (Figure 1A-D). No CSF-1R expression was found on glial fibrillary acidic protein (GFAP)-positive astrocytes or collagen synthesizing enzyme prolyl-4-hydroxylase-positive fibroblast-like leptomeningeal cells in the mixed glial cultures (Figure 1E and F respectively).

### M-CSF increases expression of microglial transcription factor PU.1

**Figure 2:**
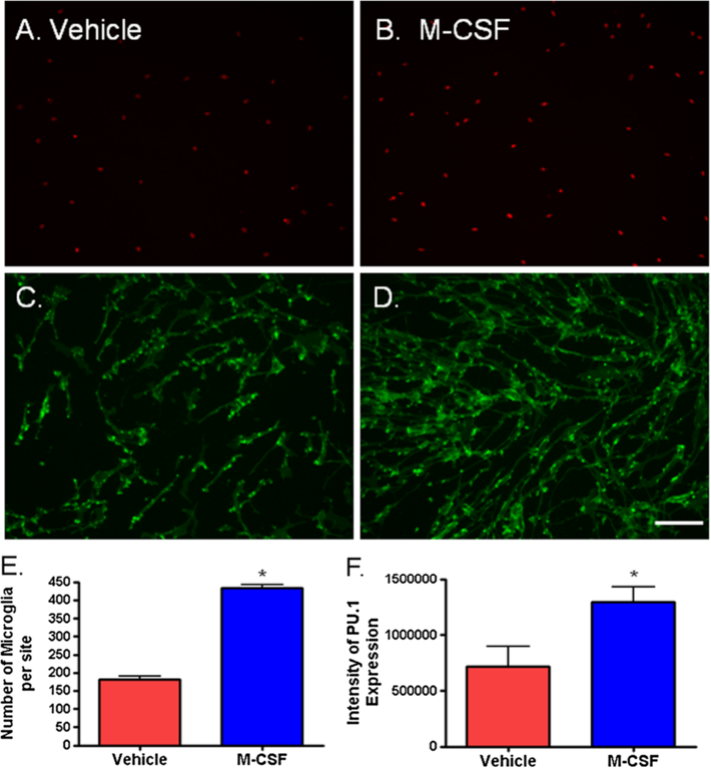
**Macrophage colony-stimulating factor (M-CSF) treatment increases the number of microglia and expression of PU.1 in primary adult human mixed glial cultures. (A)** PU.1-positive microglia (red nuclei) are present in glial cultures from human brain tissue. **(B)** There are more PU.1-positive cells with M-CSF treatment compared to vehicle treatment. The increase in microglia number is also seen by immunostaining for microglial surface receptor CD45 **(C** and **D)**. **(E)** Treatment of human glial cultures with 25 ng/ml M-CSF for 96 hours significantly increases the number of microglia present. *P* < 0.0001, n = 6. **(F)** M-CSF significantly increases the intensity of PU.1 expression (amount of PU.1 protein) in adult human microglia. *P* < 0.05, n = 6. Scale bar = 100 μm.

To assess the response of adult human microglia to M-CSF, we treated the mixed glial cultures with 25 ng/ml M-CSF for 96 hours. We used the microglial transcription factor PU.1 and the pan microglial cell surface protein CD45 to identify and count the number of microglia (Figure 2A, B and 2C, D respectively). We observed and quantified significantly more microglia in the cultures treated with M-CSF than vehicle-treated cultures (Figure 2E).

The number of PU.1-positive and CD45-positive cells was increased by M-CSF, reflecting the increase in microglial number. However, we also found a significant increase in PU.1 protein expression in microglia treated with M-CSF (Figure 2F). Thus, M-CSF increases the amount of PU.1 within microglia as well as PU.1-positive microglial number.

### M-CSF increases proliferation of adult human microglia

**Figure 3:**
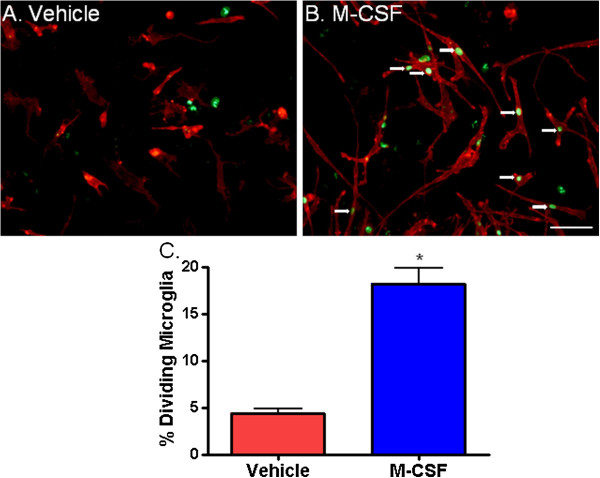
**Macrophage colony-stimulating factor (M-CSF) increases the division of adult human microglia. (A** and **B)** Immunocytochemical images of CD45 microglial cell surface marker (red) and Ki67 cell division marker (green). Adult human microglia undergo limited proliferation in culture, as demonstrated by a lack of co-expression of Ki67 by CD45-immunoreactive microglia in **(A)**. However, M-CSF treatment increases the number of dividing microglia, as shown by microglial nuclear expression of Ki67 **(B)**. Arrows indicate examples of Ki67-immunopositive microglia. **(C)** Quantification of the percentage of microglia that incorporate BrdU under control conditions and with M-CSF treatment showing a significant increase in microglial division with M-CSF. *P* < 0.01, n = 6. Scale bar = 50 μm.

To further investigate the finding that more PU.1-positive cells are present in cultures treated with M-CSF, we assessed microglial proliferation. Adult human microglia basally proliferate at a very low rate (% dividing microglia as measured by BrdU incorporation = 4.2 +/− 1.2% (mean +/− SEM; n = 6 cases)). However, treatment with M-CSF markedly increased microglial division (% dividing microglia after M-CSF treatment = 12.6 +/− 2.0% (mean +/− SEM; n = 6 cases)). M-CSF treatment resulted in a greater number of microglia expressing the cell division protein Ki67 (Figure 3A and B) and significantly increased BrdU incorporation by microglia (Figure 3C).

### M-CSF increases adult human microglial phagocytosis

**Figure 4:**
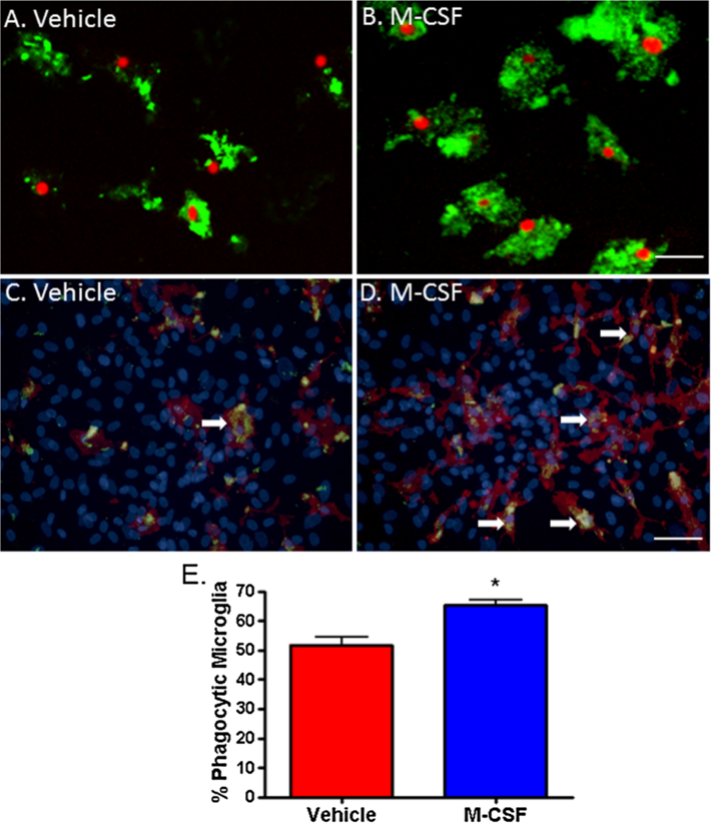
**Macrophage colony-stimulating factor (M-CSF) increases microglial phagocytosis of amyloid-β**_**1-42**_**. (A)** Microglial nuclei labeled with PU.1 (red) are surrounded by fluorescently labeled amyloid-β_1-42_ peptide (green) that has been phagocytosed by the microglia. **(B)** M-CSF treatment for 96 hours increases the amount of amyloid-β_1-42_ that has been phagocytosed by microglia. Another demonstration of this is shown in **(C)** and **(D)** where all nuclei are labeled with Hoechst and microglia are labeled with cell surface marker CD45 (red). Arrows indicate highly phagocytic microglia. **(E)** M-CSF significantly increases the percentage of microglia that undergo phagocytosis. *P* < 0.0001, n = 6. Scale bar = 50 μm.

Phagocytosis is a key innate function of microglia. Their ability to perform efficient phagocytosis is important for the healthy, as well as diseased, brain. In an assay of microglial phagocytosis of Aβ_1-42_ peptide, M-CSF-treated microglia were more phagocytic than vehicle-treated microglia (Figure 4A and C, compared to 4B and D). We found a greater proportion of highly phagocytic microglia amongst M-CSF-treated cells compared to vehicle-treated cells and the percentage of phagocytic microglia from the total PU.1-positive microglia population is significantly increased with M-CSF (Figure 4C).

### M-CSF induces a change in microglial morphology

**Figure 5:**
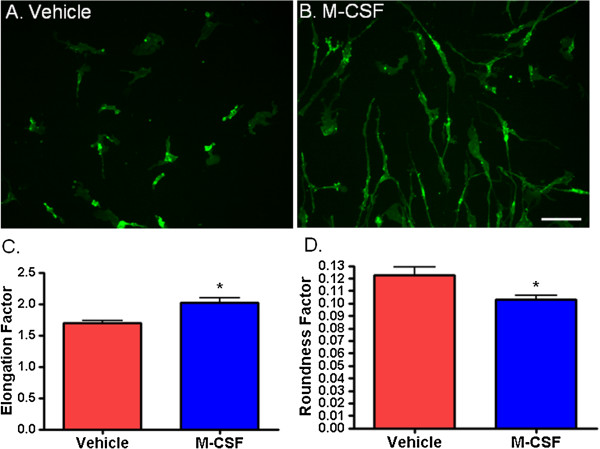
**Macrophage colony-stimulating factor (M-CSF)-treated microglia assume a rod-like shape. (A** and **B)** Immunocytochemical labeling of CD45 microglial cell surface protein shows the morphology of adult human microglia *in vitro*. **(A)** Basal adult human microglial morphology is heterogeneous - some microglia are more rounded and others are more ramified. **(B)** A shift in microglial shape to a rod-like morphology is evident after 48 hours M-CSF treatment. This significant elongation of microglia with M-CSF can be quantified using Metamorph Elliptical Form Factor (elongation; *P* < 0.001, n = 16) **(C)** and Shape Factor (roundness; *P* < 0.01, n = 16) **(D)** image analysis tools. Scale bar = 100 μm.

The morphology of untreated adult human microglia *in vitro* is heterogeneous, with cells having variable protrusions and extensions (Figure 5A). A striking effect of M-CSF on adult human microglia was a change in their morphology to elongated, slender, bipolar cells (Figure 5B). A shift in microglial shape to a ‘rod-like’ morphology is evident under a light microscope after 48 hours of M-CSF treatment and is more pronounced at 96 hours. This effect can be quantified using the Metamorph image analysis tools Elliptical Form Factor and Shape Factor. These measures of microglial shape show a significant morphological difference with M-CSF treatment (Figure 5C and D).

### M-CSF decreases HLA expression

**Figure 6:**
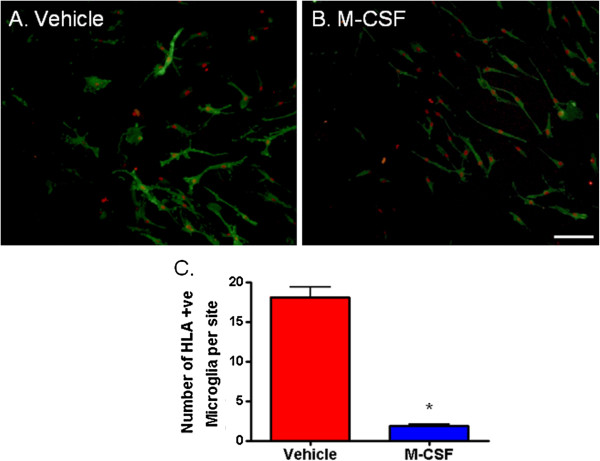
**Microglial expression of HLA-DP, DQ, DR is reduced by macrophage colony-stimulating factor (M-CSF). (A)** Representative image of basal HLA expression (green) in cultured adult human microglia. HLA expression is highly variable between cases and is expressed by some, but not necessarily all, microglia (PU.1-positive nuclei, red). **(B)** Following 96 hours exposure to M-CSF, the level of microglial HLA expression was found to be decreased compared to basal levels. **(C)** Quantification of the effect of M-CSF on HLA expression shows a significant reduction in the number of microglia expressing HLA. *P* < 0.0001, n = 18. Scale bar = 100 μm.

To further characterize the phenotype of M-CSF-treated microglia and elucidate the functional significance of the M-CSF-induced change in morphology, we looked at the levels of HLA-DP, DQ, DR expressed by microglia. Human microglial expression of HLA-DP, DQ DR is inducible and is highly variable between cases (from a sample of ten cases; four cases had high basal microglial expression of HLA-DP, DQ, DR, three cases had moderate expression and three cases had no expression). In the majority (six out of seven) of cases which had basal microglial HLA-DP, DQ, DR expression, M-CSF treatment was found to decrease the level of expression (Figure 6A and B). Quantification of microglial HLA-DP, DQ, DR expression clearly shows a significant reduction with M-CSF (Figure 6C).

On the other hand, no change in CD45 expression was observed with M-CSF treatment (Figure 2). When the morphological analysis quantification tools were applied to CD45-immunopositive and HLA-DP, DQ, DR-immunopositive microglia under basal conditions, there was no difference in Elliptical Form Factor or Shape Factor morphology parameters.

### M-CSF increases microglial expression of C/EBPβ transcription factor and DAP12 adaptor protein

**Figure 7:**
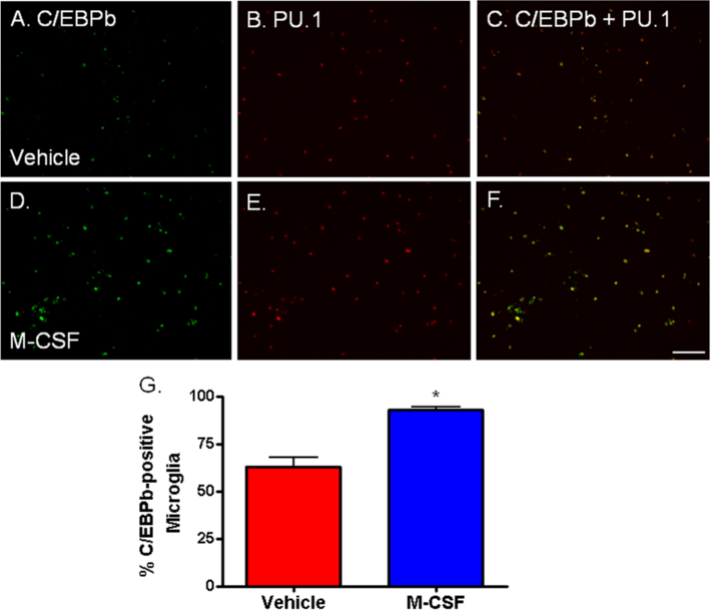
**Macrophage colony-stimulating factor (M-CSF) increases adult human microglial expression of C/EBPβ. (A**-**C)** C/EBPβ is expressed by PU.1-immunopositive adult human microglia in mixed glial cultures. **(D**-**F)** M-CSF increases the intensity of C/EBPβ labeling in adult human microglia and the number of microglia which express C/EBPβ. **(G)** Quantification of microglial C/EBPβ expression showing that M-CSF increases the percentage of microglia which express C/EBPβ. *P* < 0.001, n = 12. Scale bar = 100 μm.

To investigate the molecular events precipitated by M-CSF treatment, we looked for changes in factors known to be associated with PU.1. From the CCAAT enhancer-binding protein (C/EBP) transcription factor family, we detected an increase in C/EBPβ expression within microglia in mixed glial cultures with M-CSF treatment (Figure 7). A quantifiable increase in the percentage of PU.1-immunoreactive microglia expressing C/EBPβ was found following M-CSF treatment (Figure 7G). While the percentage of CD45-positive microglia expressing PU.1 (100%) did not change with M-CSF treatment, the percentage of CD45-positive microglia expressing C/EBPβ was increased with M-CSF treatment. C/EBP-β is known to interact with PU.1 and may be associated with the increase in PU.1 expression levels (Figure 2F).

**Figure 8:**
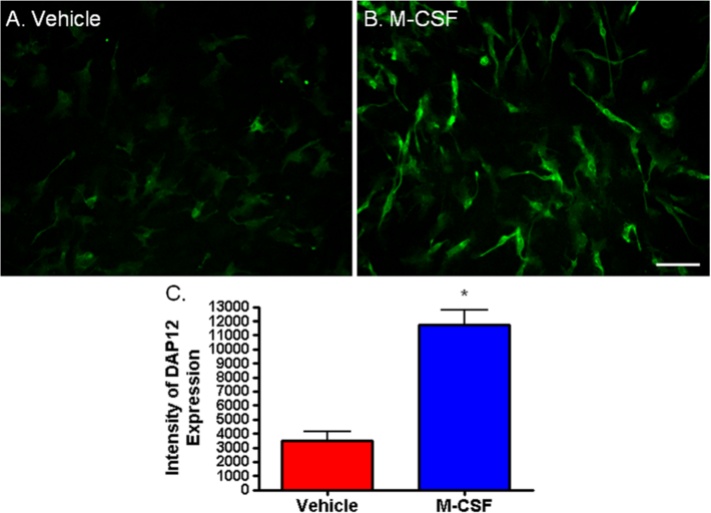
**Macrophage colony-stimulating factor (M-CSF) increases adult human microglial expression of DAP12. (A)** DAP12 monocyte/microglial adaptor protein is expressed by human microglia isolated from biopsy tissue. **(B)** Treatment of microglia with M-CSF increases their expression of DAP12 as seen by increased intensity of immunochemical staining. **(C)** Quantification of DAP12 staining intensity shows a significant increase in DAP12 expression with M-CSF treatment. *P* < 0.0001, n = 8. Scale bar = 100 μm.

DAP12 is a myeloid adapter protein found in microglia in the human brain [42]. Signaling through DAP12 has been associated with the PU.1 transcription factor [22]. We found that M-CSF increased expression of DAP12 in adult human microglia such that, concurrent with the increase in PU.1 and C/EBPβ, there is a significant, quantifiable increase in intensity of DAP12 staining (Figure 8).

### M-CSF increases microglial expression of M-CSF and IGF-1 receptors

**Figure 9:**
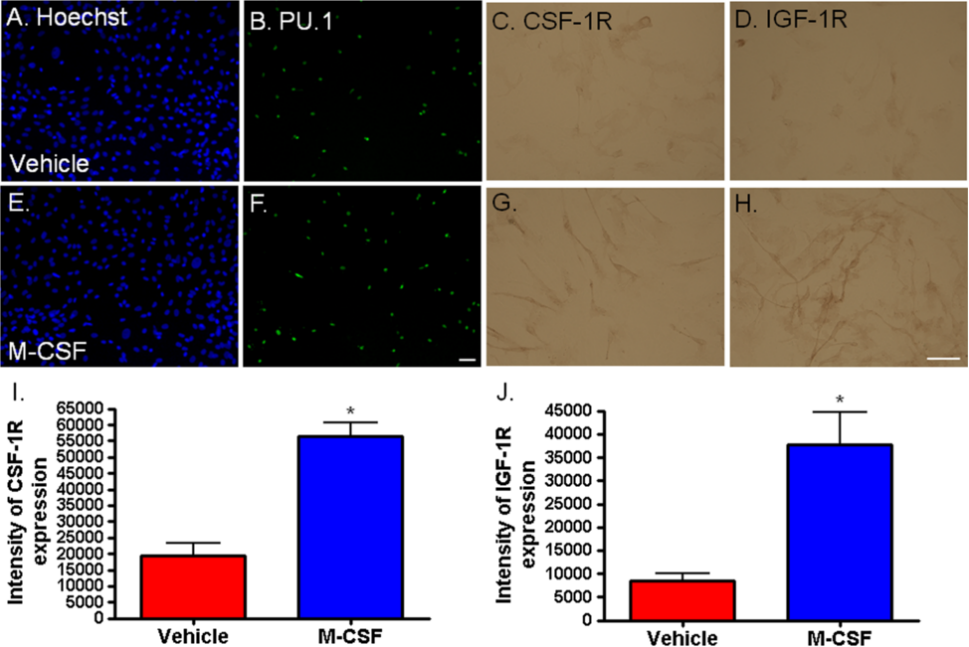
**Macrophage colony-stimulating factor (M-CSF) increases adult human microglial expression of CSF-1R and IGF-1R.** M-CSF treatment does not significantly alter the total number of Hoechst-labeled nuclei in human adult mixed glial cultures **(A** and **E)**, but it does increase the number of microglia **(B** and **F)**, as shown by labeling for the microglial transcription factor PU.1. Together with an increase in microglia number, M-CSF produces an increase in microglial expression of its receptor CSF-1R **(C** and **G)** and the receptor for IGF-1 **(D** and **H)**. A significant increase in intensity of receptor labeling is found for both CSF-1R **(I**; *P* < 0.0001, n = 8**)** and IGF-1R **(J**; *P* < 0.01, n = 8**)** following M-CSF treatment. Scale bar = 50 μm.

As shown in Figure 1D, adult human microglia express the M-CSF receptor, CSF-1R, *in vitro*. Furthermore, on exposure to M-CSF (25 ng/ml) for 96 hours, microglial expression of CSF-1R was increased (Figure 9C and G). We see again an increase in microglial number after exposure to M-CSF but we also see an increase in intensity of CSF-1R staining (Figure 9I).

We also assessed levels of another mitogenic growth factor receptor, Insulin-like growth factor 1 receptor (IGF-1R), which has previously been reported to be linked to M-CSF signaling [43]. M-CSF treatment co-incidentally increased microglial expression of both IGF-1 (Figure 9D, H and J) and M-CSF receptors (Figure 9C, G and I).

## Discussion

M-CSF has numerous interesting effects on adult human microglia, many of which may have significance for a range of neurological diseases.

The numbers of PU.1 and CD45 immunopositive cells were increased by M-CSF treatment and we have shown that this is at least partially through an increase in microglial division. This division effect was immediately apparent as adult human microglia do not frequently divide when cultured in basic medium (DMEM/F12 + FBS + PSG). This observation is in line with previous studies noting a proliferation effect with M-CSF for human fetal microglia [14], and up-regulation of M-CSF following axotomy of the rat facial nucleus which triggered microglia to proliferate [17]. Many myeloid cells have been shown to have M-CSF growth dependence [44]. It may be that adult human microglia require M-CSF for division, but not for survival, *in vitro*. The proportion of microglia that undergo division is specific to each individual patient. However, the result of increased microglial division with M-CSF treatment is consistent between cases.

It has previously been shown that microglia in the rat brain, as well as the BV2 rodent microglial cell line, constitutively express a high level of PU.1 in both the ‘normal’ and post-injury state [45]. We have recently shown that microglia in the adult human brain also express the transcription factor PU.1 [23]. Previous studies have demonstrated that PU.1 regulates the M-CSF receptor [25]. We have found here that PU.1 also acts down-stream of M-CSF signaling, as PU.1 protein expression is increased in microglia following M-CSF treatment (Figure 2). PU.1 has been shown to be involved in M-CSF-dependent proliferation of mouse bone-marrow macrophages [24] and thus it is likely that PU.1 is involved in the M-CSF-induced proliferation of adult human microglia, as well as many of the other processes discussed below.

Phagocytosis is an important innate function of microglia as part of their role to respond to cell injury and regulate the extracellular environment. We found that M-CSF increased adult human microglial phagocytosis of Aβ_1-42_ peptide by microglia from different cases, regardless of the starting level of phagocytosis. In the context of Alzheimer’s disease it is thought that microglia may be helpful in clearing the brain of extracellular deposits of Aβ_1-42_ protein [46,47]. M-CSF has been shown to be beneficial in Alzheimer’s disease mouse models and one mechanism that may be responsible for this effect is increased phagocytosis of Aβ_1-42_[30]. In another recent study using human microglia, M-CSF treatment (in combination with IL-4 and IL-13) increased microglial phagocytic ability for myelin debris compared to microglia stimulated with a combination of GM-CSF, IFNy and LPS [48].

We have previously found that PU.1 is necessary for basal phagocytosis in human adult microglia [23]. DAP12 also appears to be involved in the phagocytic process [49] and associates with proteins which have been found to play a role in phagocytosis, for example CD68, TREM2 and SIRPB1 [42,50]. Given the M-CSF-induced increases in PU.1 and DAP12 expression, it is likely that these proteins are involved in M-CSF-dependent phagocytosis in adult human microglia.

Another prominent effect of M-CSF on adult human microglia is their change in morphology to bipolar, elongated (‘ramified’) cells. Microglial morphology is presumed to relate to their function, although exactly how is currently unclear. Round ‘amoeboid’ microglia are traditionally viewed as activated, inflammatory microglia. The M-CSF-induced morphology change could be a sign of microglia being ‘primed’ towards a particular activation state. However, microglial phenotype is multifaceted and M-CSF-induced elongation doesn’t prohibit microglia from changing morphology when exposed to other molecules, for example Aβ_1-42_ which induces changes to their cytoskeletons necessary for phagocytosis. Even though the M-CSF-treated microglia are more rod-shaped and ‘ramified’ than vehicle-treated microglia, they have an increased propensity to phagocytose compared to control microglia of heterogeneous morphology.

Durafourt *et al*. (2012) used the same concentration of M-CSF as part of an ‘alternative’ macrophage/microglia activation polarizing protocol. Whereas macrophages under these conditions had more extended processes, no change was observed for microglia. It may be that the CD45 marker of microglia used here is better for observing morphological differences, or that their addition of IL-4 and IL-13 reduced the morphology effect seen in microglia [48]. The M-CSF-induced ‘rod’-like effect has also been noted for human fetal microglia [14].

Elongated rod-shaped microglia have also been reported *in situ* in rodent models of neurological injury including ischemia [51,52]. Graeber (2010) has reported on microglial rod cells observed in human brain tissue associated with cognitive symptoms and psychopathologies [10]. Rod-shaped, elongated microglia have been reported in the Huntington’s disease cortex [6] and in subacute sclerosing panencephalitis, Alzheimer’s disease and Wilson’s disease brains [53]. In concordance with our findings, Wierzba-Bobrowicz *et al*. (2002) also noticed proliferating cell nuclear antigen co-labeling with rod microglia and reduced expression of HLA compared to more rounded microglia [53].

Rod-shaped microglia are relatively under investigated [10] and our study presents some of the first functional results relating to the findings of rod-microglia in human brain tissue. If M-CSF-treated adult human microglia are indeed *in vitro* correlates of the ‘rod’ microglia reported in diseased adult human brain tissue, this will provide an invaluable tool for investigation into this particular microglial phenotype. Furthermore, our method for quantifying microglial morphology is a quick, high throughput and unbiased way of assessing these changes, compared to laborious and subjective quantification by eye.

To determine the functional relevance of the morphology change and to look further at the activation state of these M-CSF-exposed microglia, we investigated whether their expression of HLA was affected. HLA-DP, DQ, DR is an inducible protein and we find that its expression by microglia varies widely between cases. We asked whether this inducible expression of HLA-DP, DQ, DR was modulated by M-CSF and found that, despite an increased number of microglia, fewer microglia express high levels of HLA-DP, DQ, DR with M-CSF. HLA-DP, DQ, DR expression by microglia is often taken to represent an ‘activated’ or inflammatory microglial phenotype. Our results suggest that M-CSF-treated microglia may be alternatively activated and have reduced antigen presentation capacity.

This M-CSF effect of reduced HLA-DR has also been noted for human fetal microglia [14]. Furthermore, expression of HLA class II molecules was noted to be less intensive on the surface of microglial rod cells compared to neighboring ramified microglia in neurologically diseased human brain tissue [53]. In the different context of mouse monocytic precursor cells, Henkel *et al*., (2002) found that M-CSF-induced maturation increased MHC class II expression with IFNy [22]. This finding indicates that the effect of M-CSF on HLA may be dependent on cell differentiation stage and/or species. Conversely, Melief *et al*. (2012) have recently shown that the ‘alternative’ activating cytokine IL-4 increases HLA-DR mRNA expression whilst also inducing an elongated morphology in adult human microglia [54]. Overall, these results demonstrate the wide phenotypic diversity of microglia.

An HLA-DQ-derived peptide has been found to have anti-proliferative effects [55], suggesting that the decrease in HLA-DP, DQ, DR seen in the present study may be mechanistically related to the increase in microglial proliferation also seen with M-CSF. Thus decreased HLA may not only have implications for antigen presentation, but perhaps for other cellular functions like proliferation.

Of particular interest in our study is that an up-regulation of PU.1 with M-CSF correlates, in general, with a less activated microglial phenotype (for example, reduced HLA-DP, DQ, DR expression), although phagocytosis was stimulated. This contrasts with a recent report by Ponomarev *et al*. showing that increased PU.1 was associated with an activated microglial phenotype in rodents [56]. The most likely reason for these different results is species differences, highlighting the importance of studying adult human brain microglia.

To decipher the mechanisms by which the M-CSF-induced phenotypic changes occur in microglia, we looked for changes in factors known to be associated with the transcription factor PU.1.

CCAAT enhancer-binding protein (C/EBP) transcription factors are expressed throughout the body including the brain [45,57]. The C/EBP family has already been shown to have a number of species-specific regulation processes and expression patterns [57]. From the C/EBP transcription factor family we detected an increase in C/EBPβ expression within microglia in human mixed glial cultures treated with M-CSF.

C/EBPβ plays numerous roles in activation and differentiation of macrophages [57]. It has been reported to have a role in inflammatory processes in rodents [58-60] and may play a role in differentiated macrophage morphology [61]. Although our studies find increased C/EBPβ along with increased microglial proliferation, C/EBPβ has been reported to inhibit proliferation of human THP-1 monocytic cells and murine macrophage-like cells [61]. However, other reports have shown that C/EBPβ can promote proliferation [57] and furthermore, be involved in M-CSF-directed mechanisms in tumors [62] and HIV infection [63]. C/EBP transcription factors including C/EBPβ can transactivate the CSF-1R promoter in mammalian cell line COS-7 cells [64], and could also be involved in the increase in CSF-1R expression observed in our experiments. C/EBPβ has recently been demonstrated in human spinal cord tissue from amyotrophic lateral sclerosis patients, but rarely in controls, co-localized with the microglial marker CR3 [58].

C/EBPβ forms heterodimers with members of its own family and interacts with several other transcription factors [57] including PU.1 [65,66]. M-CSF-mediated enhanced PU.1 and C/EBPβ transcription factor protein expression have also been reported for a murine myeloblastic cell line [67]. Furthermore, co-expression of PU.1 and C/EBPβ in fibroblasts can induce a macrophage phenotype [44]. PU.1 and C/EBPβ transcription factors together may be responsible for many of the M-CSF induced effects we find in adult human microglia.

DAP12 is a myeloid adapter protein found in microglia in the brain. We found that M-CSF treatment of adult human microglia increased their DAP12 expression. It has been shown to be involved in M-CSF-induced proliferation and survival of mouse bone marrow-derived macrophages [68]. The concurrent increase in DAP12 protein expression with increased adult human microglia number and proliferation suggest a role for DAP12 in this M-CSF-induced mechanism. DAP12 could also be involved in the process of phagocytosis as primary mouse microglia transduced with mutant DAP12 have reduced phagocytic ability [50].

Henkel *et al*. (2002) demonstrated an upregulation of DAP12 in PU.1-rescued monocytic precursor cells and Weigelt *et al*. (2007) have shown that DAP12 expression is dependent on PU.1 via a binding site in the DAP12 proximal promoter [22,49]. Thus the M-CSF-induced increase in DAP12 expression may be directly mediated by the increase in PU.1. Furthermore, the role of M-CSF in determining PU.1 and DAP12 expression in microglia may have implications for many neurological diseases [69].

CSF-1R expression is shown here to be restricted to microglia, and not detected on other cell types in our cultures, both basally and with M-CSF treatment. In addition, we found that M-CSF increased the expression of its receptor on microglia. The increase in PU.1 found with M-CSF treatment is likely to directly increase CSF-1R levels as it has been reported to regulate *c-fms* transcript expression [25]. Yamamoto *et al*. (2010) have also found that M-CSF increases microglial CSF-1R expression in the context of the rat axotomized facial nucleus [17].

Coincidentally, M-CSF treatment also increased microglial expression of the IGF-1 receptor. There have been a number of associations previously reported between the growth factors M-CSF and IGF-1 [43]. Both factors are mitogenic, play critical roles in development and have similar regulation mechanisms. In a study of mouse macrophage tumor cells, Wessells *et al*. (2004) found C/EBPβ to have a critical role in cell survival, in part by regulating expression of IGF-I. Furthermore, M-CSF was found to compensate for IGF-1 and could rescue IGF-1-deficient cells [70]. The overlapping functions of these ligands may explain the simultaneous increase in both the CSF-1 and IGF-1 receptors in response to M-CSF.

Another CSF-1R ligand, IL-34, has recently been discovered and is expressed throughout the body, including the brain [71]. Like M-CSF, IL-34 is involved in human monocytic proliferation and viability [71,72] but its biological activity and signal activation are not identical to that of M-CSF [72]. Furthermore, it has been suggested that the *in vivo* role of IL-34 may differ between rodents and humans [72], and research into the effects of this cytokine on microglia in the human brain are warranted.

## Conclusions

M-CSF dramatically influences the phenotype of adult human microglia. We have found that M-CSF has many fascinating effects on these human brain cells and has an important role in determining microglial phenotype and function in the context of the adult human brain. However, the *in vivo* implications of these results are yet to be determined. Therapeutically, it would be desirable to have the ability to modulate microglial phenotype towards a protective role. To this end, M-CSF-induced human microglia are worthy of further investigation.

## Abbreviations

Aβ1-42: Amyloid- β_1-42_ protein; BrdU: 5-bromo-2′-deoxyuridine; C/EBP: CCAAT enhancer-binding protein; CSF -1R: Colony-stimulating factor-1 receptor/M-CSF receptor; DAB: 3,3′-diaminobenzidine tetrahydrochloride; FBS: Fetal bovine serum; GFAP: Glial fibrillary acidic protein; HLA: Human leukocyte antigen; IGF-1: Insulin-like growth factor-1 receptor; IL: Interleukin; M-CSF: Macrophage colony-stimulating factor; PFA: Paraformaldehyde; SEM: Standard error of the mean.

## Competing interests

The authors have no competing interests.

## Authors’ contributions

AMS, HMG and MD conceived and designed the experiments. AMS performed cell isolation and cell culture experiments, immunocytochemistry, image acquisition and analysis. AMS and MD interpreted data and wrote the manuscript. RLO, PSB, EWM, MAC and RLMF contributed materials and revised the manuscript. All authors read and approved the final manuscript.
